# Supplementary material for: E47 modulates hepatic glucocorticoid action
Source: Nat Commun. 2019 Jan 18;10:306. doi: 10.1038/s41467-018-08196-5 (PMC6338785; doi:10.1038/s41467-018-08196-5)
Supplement: Supplementary file 1 — Supplementary Information [file 41467_2018_8196_MOESM1_ESM.pdf]

Supplementary Figures and Tables

Hemmer et al.

E47 mediates hepatic glucocorticoid action

Supplementary Figure 1: E2A expression and genomic binding in mouse liver.

A

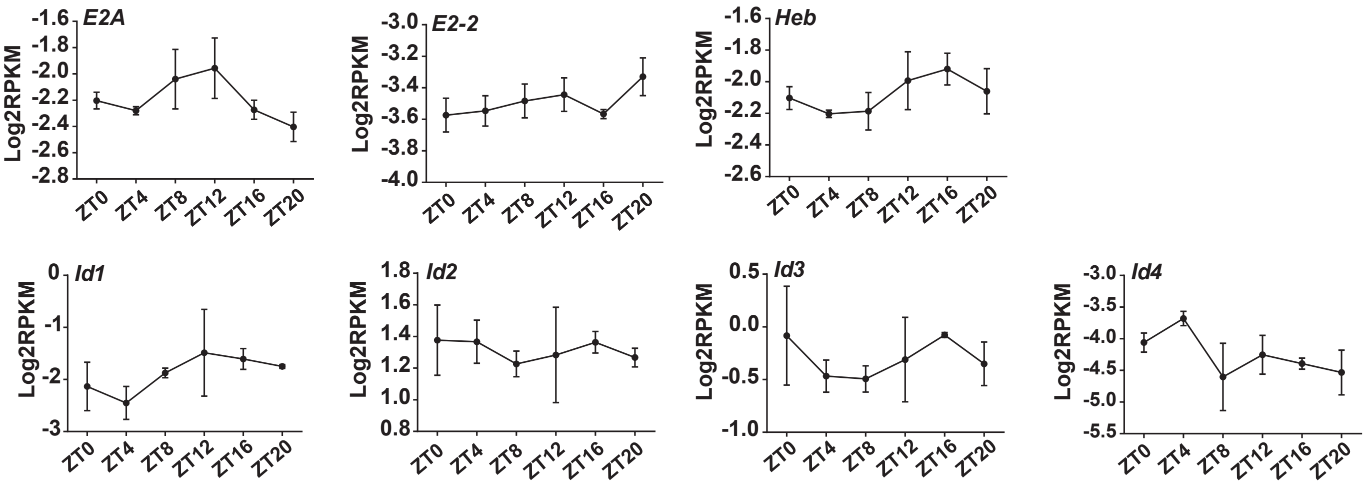

B

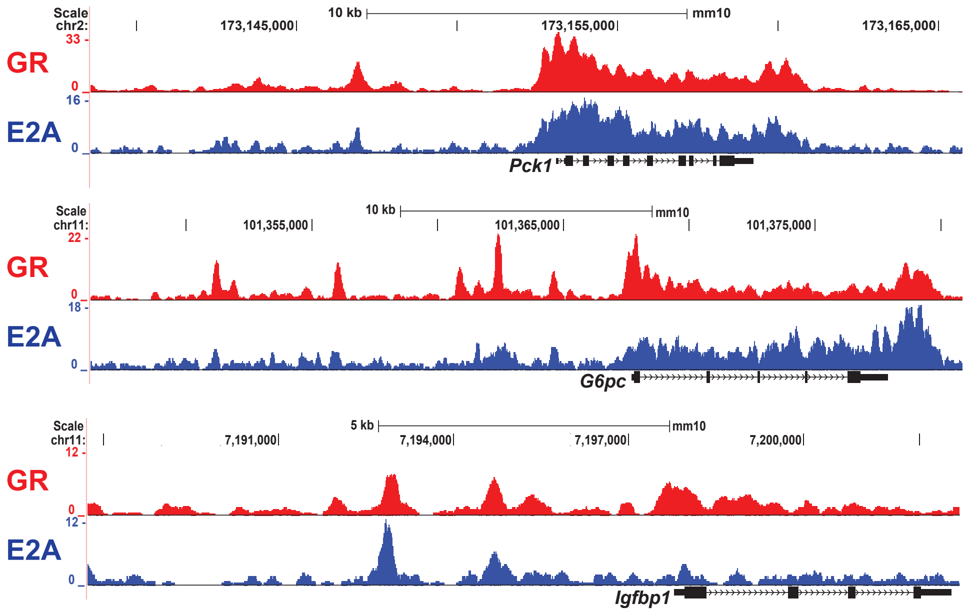

C

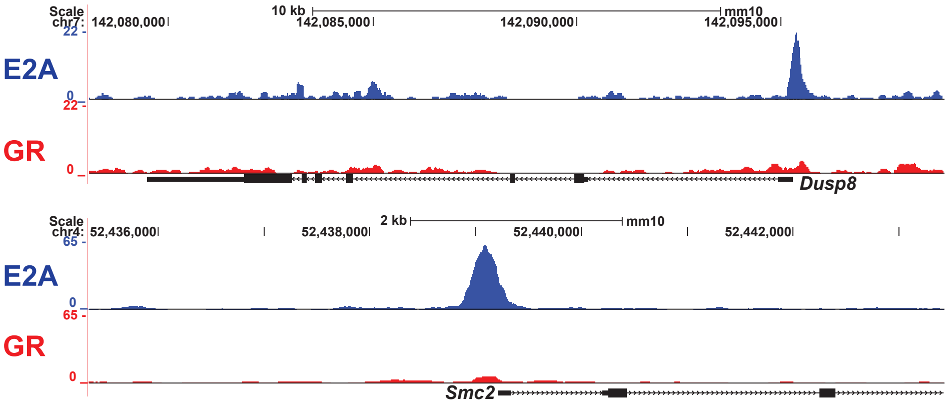

D

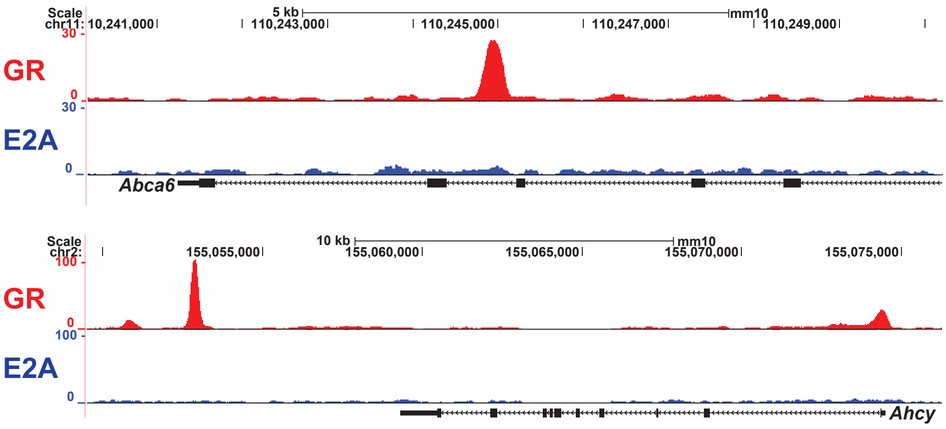

A: RNA-Seq data from WT livers at 6 time-points around the clock (ZT0= 7am; ZT12= 7pm). Expression of E-Box proteins (E2A, E2-2, Heb) and ID-Proteins (Id1, Id2, Id3, Id4) is displayed as mean Log2RPKM ±SEM, n= 4. **B:** Representative ChIP-Seq tracks displaying co-occupancy of GR and E2A at metabolic promoters and enhancers in WT livers (red= GR; blue=E2A) **C:** Representative ChIP-Seq tracks showing E2A-specific occupancy at hepatic promoters/enhancers, and functional annotation of E2A-specific target genes by gene ontology (GO) analysis, assigning ChIP peaks to the nearest coding gene. **D:** Analogous representation of GR-specific occupancy and functional annotation of GR-specific targets by GO analysis (red= GR; blue=E2A).

GO: Biological process	FDR Q-value
Cellular amino acid metabolic process	1.14e-2
Carboxylic acid catabolic process	2.23e-2
Inactivation of MAPK activity	2.14e-2

Examples:
Got1, Idh1, Acad11, Dusp8, Smc2

GO: Biological process	FDR Q-value
Small molecule metabolic process	1.52e-42
Carboxylic acid metabolic process	9.43e-37
Lipid metabolic process	6.99e-28
Fatty acid metabolic process	5.94e-20
Cellular carbohydrate metabolic process	9.28e-12

Examples:
Abca6, Acot11, Elovl5, Dgat2, Cpt1α

Supplementary Figure 2: Phenotypic characteristics and gene expression data from *E47-/-* mice.

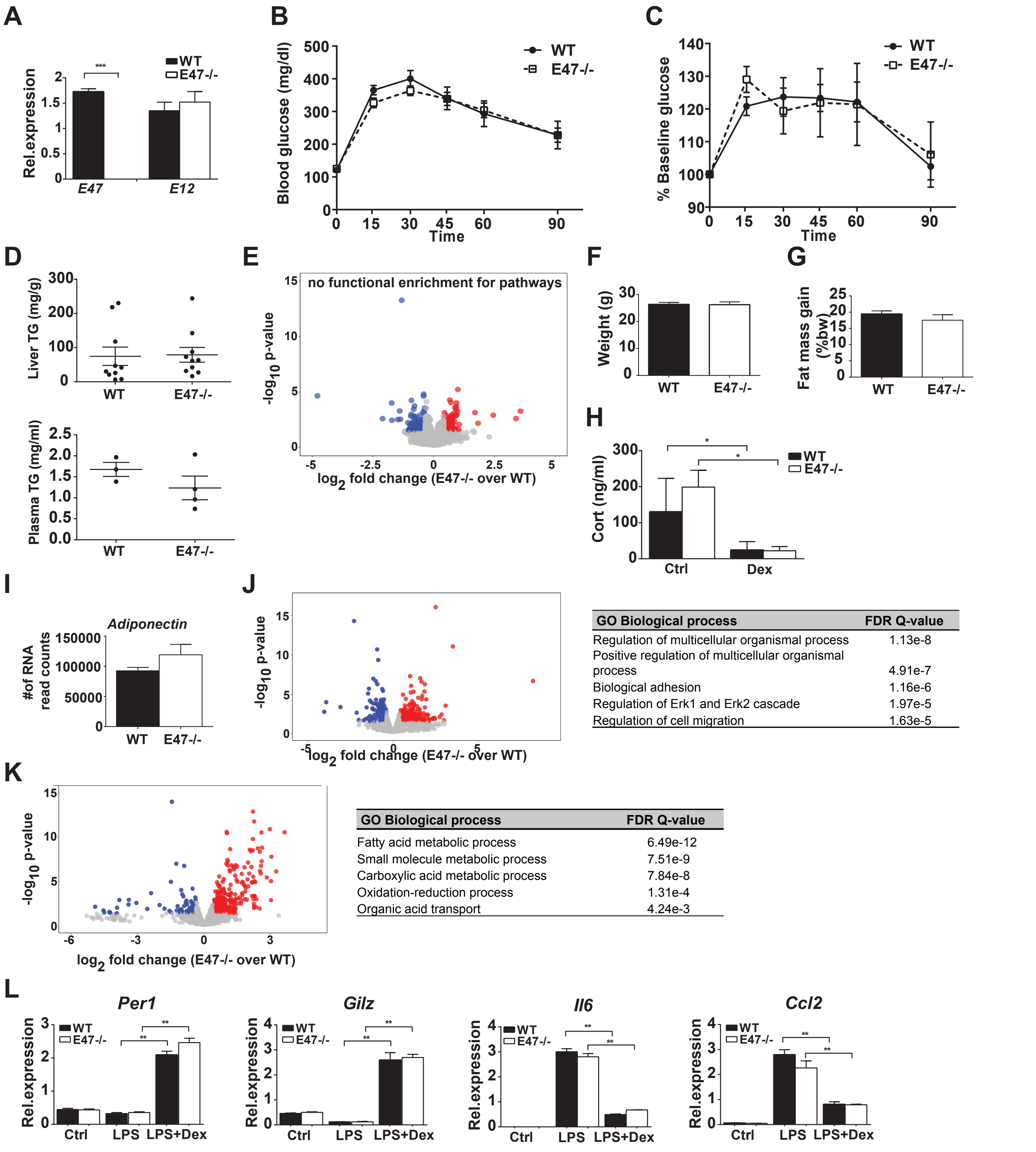

GO Biological process	FDR Q-value
Regulation of multicellular organismal process	1.13e-8
Positive regulation of multicellular organismal process	4.91e-7
Biological adhesion	1.16e-6
Regulation of Erk1 and Erk2 cascade	1.97e-5
Regulation of cell migration	1.63e-5

GO Biological process	FDR Q-value
Fatty acid metabolic process	6.49e-12
Small molecule metabolic process	7.51e-9
Carboxylic acid metabolic process	7.84e-8
Oxidation-reduction process	1.31e-4
Organic acid transport	4.24e-3

A: qRT-PCR of E47 and E12 expression in mouse livers after Dex treatment, normalized to U36b4. Data are expressed as mean ± SEM, (***) P<0.0001, Student's t-test, n=10 (WT) & 7 (E47-/-). **B:** i.p. GTT and **C:** i.p. PTT in untreated E47 -/- and WT mice. Data were analyzed by ANOVA and Bonferroni's multiple comparison test and are shown as mean ± SEM, n= 9 (WT) & 15 (E47-/-) for GTT; n= 6 (WT) & 7 (E47-/-) for PTT. **D:** Liver and plasma triglycerides in untreated mice. Data are mean ± SEM, n= 10 per genotype (liver); n= 3 (WT) & 4 (E47-/-) (plasma). **E:** Volcano plot and GO analysis of differentially expressed genes (blue= down-; red=up-regulated) in untreated mouse livers (FC 1.3, P<0.05, n= 5 (WT) & 6 (E47-/-)). For GO analysis a base mean cutoff >200 was used. **F:** Body weight of untreated E47-/- and WT mice. Data are mean ± SEM, n= 7 (WT) & 9 (E47-/-). **G:** Fat mass gain as percentage of body weight after Cort treatment. Data are mean ± SEM, n=8 (WT) & 11 (E47-/-). **H:** Plasma corticosterone of mice 6hrs after injection of 1mg/kg Dex or vehicle. Data are mean ± SEM, UT: n=7 per genotype; Dex-injected: n=6 (WT) & 5 (E47-/-). **I:** Average number of normalized NGS read counts for Adipoq in Dex-treated white adipose tissue. Data are mean ± STDEV, n=2 per genotype. **J:** Volcano plot and GO analysis of differentially expressed genes (blue= down-; red=up-regulated) in white adipose tissue (WAT) and skeletal muscle (SM) (**K**) of E47 -/- mice on Dex (FC 1.3, P<0.05); For GO analysis a base mean cutoff >200 was used; SM: n=2 (WT) & 3 (E47-/-); WAT: n= 2 per genotype. **L**: qRT-PCR in macrophages isolated from E47 -/- and WT mice and treated with LPS or LPS+Dex, normalized to U36b4; data are mean ± STDEV (**) P<0.01, (***) P<0.001, Student's t-test, n=2 per genotype.

Supplementary Figure 3: Measurements in *E47* liver specific mutant mice.

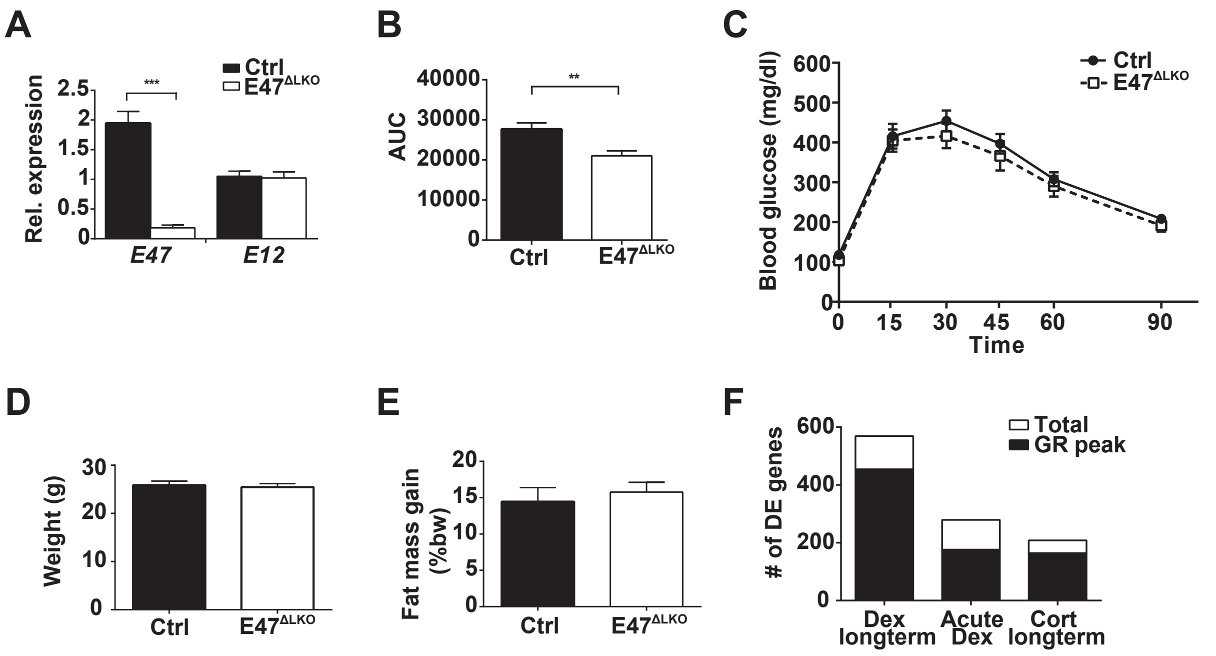

A: qRT-PCR of E47 and E12 expression in livers after Dex treatment, normalized to U36b4. Data are mean ± SEM, (***) P<0.0001, Student's t-test, n= 7 (Ctrl) & 5 ($E47^{\Delta LKO}$). **B:** Area under the curve (AUC) of GTT (Fig. **3A**). Student's t-test was performed on AUC, (**) P<0.01 **C:** i.p. GTT in untreated $E47^{\Delta LKO}$ and control mice. Data were analyzed by ANOVA and Bonferroni's multiple comparison test and are mean ± SEM, n=10 (WT) & 11 ($E47^{\Delta LKO}$). **D:** Body weight of untreated $E47^{\Delta LKO}$ and control mice. Data are mean ± SEM, n=7 per genotype. **E:** Fat mass gain as percentage of body weight on Cort. Data are mean ± SEM, n=8 per genotype. **F:** Number of genes differentially expressed in E47 mutant mice with a nearby GR ChIP peak (see Fig. **3G**).

Supplementary Figure 4: Expression levels and binding in mutant mice.

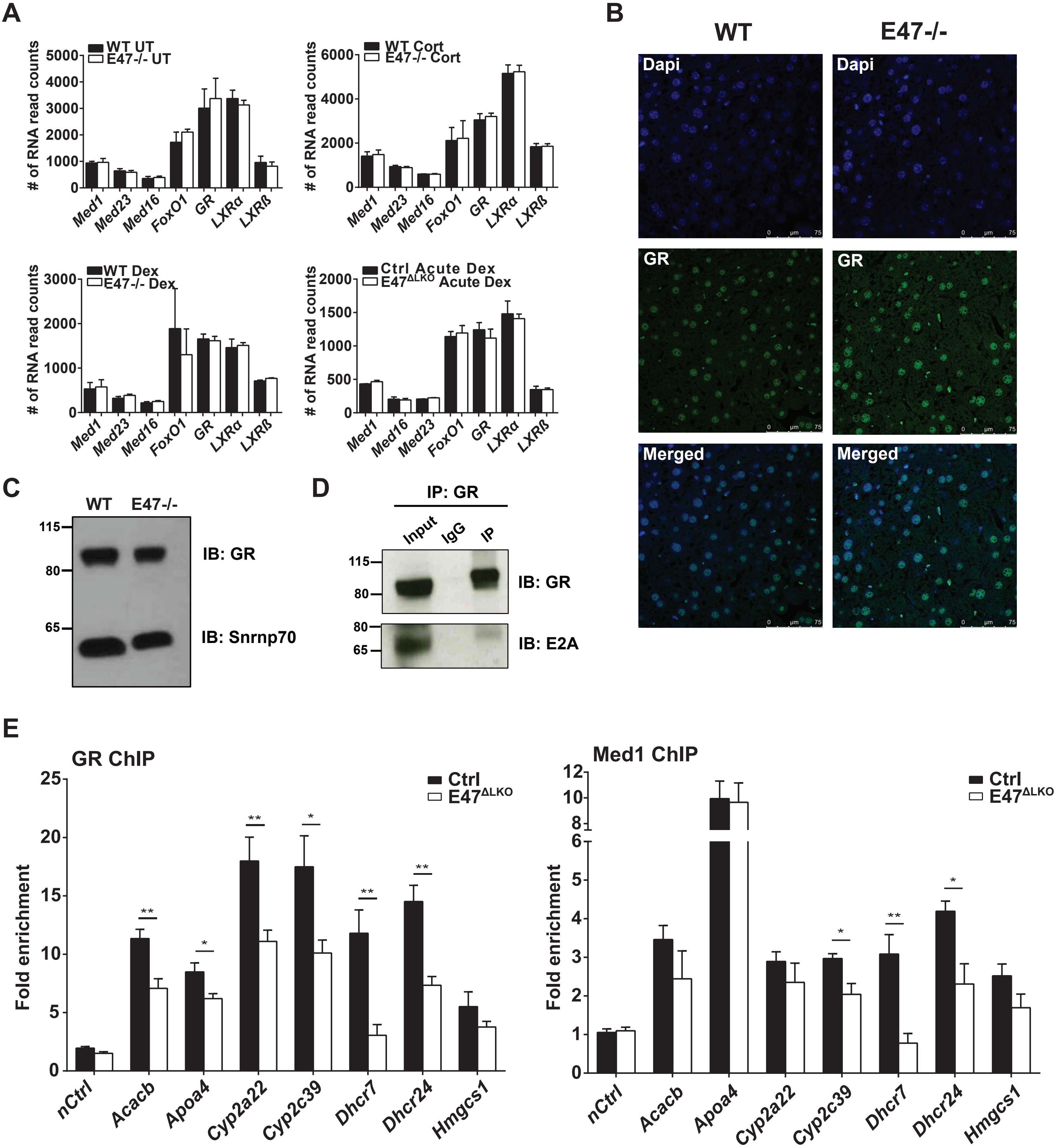

A: Number of normalized read counts for Med1, Med23, Med16, Foxo1, GR, Lxrα and Lxrβ from RNA-Seq data in livers either untreated (UT), Cort or Dex treated or Dex-injected (Fig. 2&3 and Suppl. Fig. 2). Data are mean ±STDEV. **B:** Immunostaining for GR in Dex-injected E47-/- and WT livers co-stained with DAPI, 63x magnification (75µm), confocal microscopy. **C:** Western blot of nuclear extracts from Dex-treated WT and E47-/- livers. Immunoblot (IB) for GR and Snrp70. **D:** Co-IP of GR and E2A in Dex-treated WT livers. Immunoblot (IB) for 30% input and IP against GR. **E:** ChIP-qPCR in Cort-treated $E47^{\Delta LKO}$ and control livers shows binding of GR & Med1 at metabolic promoters/enhancers. Data are shown as fold enrichment over IgG (n= 4 (Ctrl) and 6 ($E47^{\Delta LKO}$). Data are mean ±SEM, (*) P<0.05, (**) P<0.01, Student's t test.

Supplementary Figure 5: Data from fasted *E47* mutants.

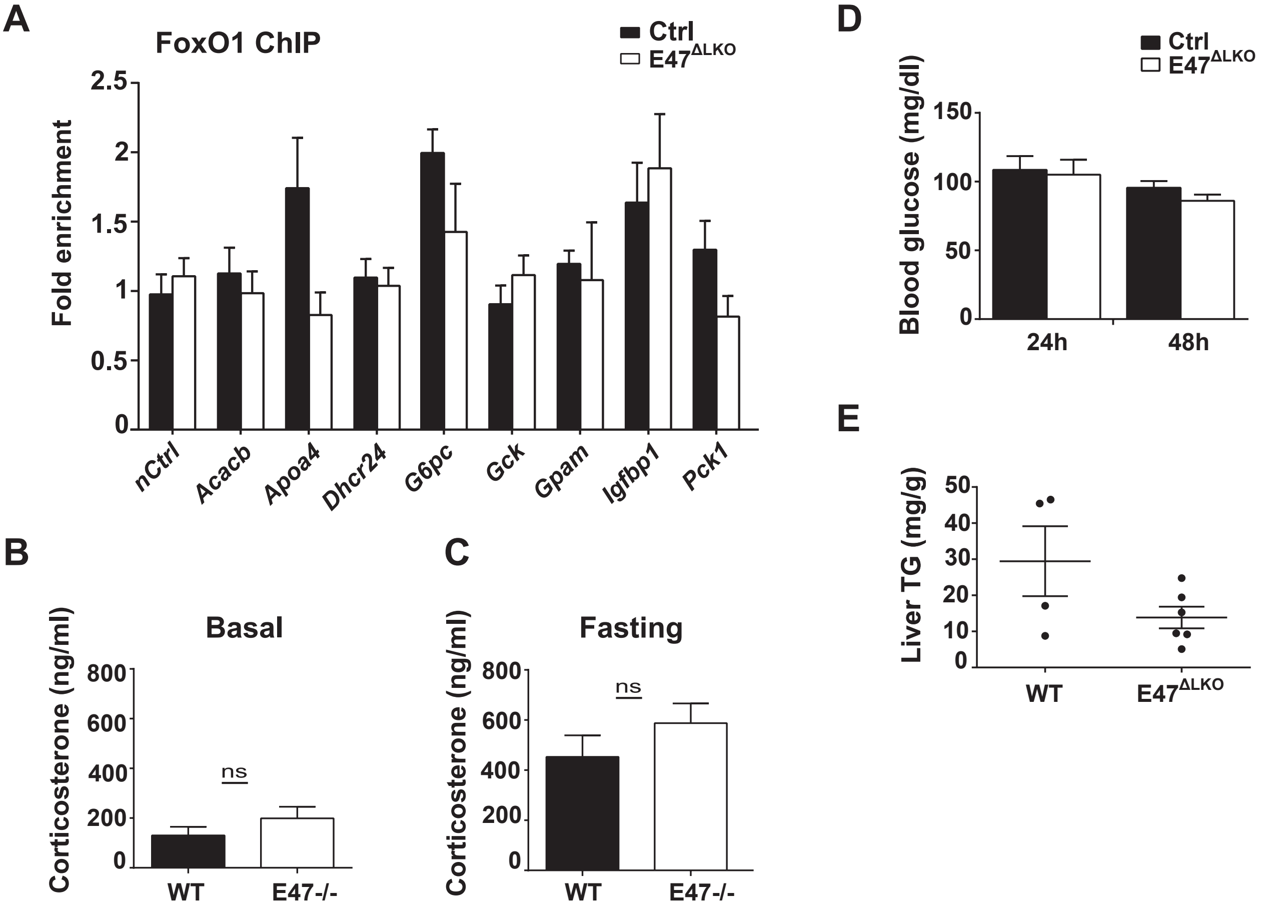

A: ChIP-qPCR in fasted E47ΔLKO and control livers shows binding of Foxo1 at metabolic promoters/enhancers. Data are shown as fold enrichment over IgG, n= 6 (Ctrl) and 4 ($E47^{\Delta LKO}$). Data are mean ±SEM. **B:** Plasma corticosterone levels of untreated E47-/- mice and wildtype littermates, n=7 per genotype (Data taken from Supplementary Figure 2H). **C:** Plasma corticosterone levels of 48h-fasted E47-/- mice and wildtype littermates, n=8 (WT) & 5 (E47-/-). **D:** Blood glucose levels of E47 $^{\Delta LKO}$ mice and control littermates measured at 24hrs and 48hrs of fasting. Data are mean ± SEM, n=4 (Ctrl) & 5-6 ($E47^{\Delta LKO}$). **E:** Liver triglycerides in 48h-fasted E47-/- mice and wildtype littermates. Data are mean ± SEM, n= 4 (Ctrl) & 6 ($E47^{\Delta LKO}$).

Supplementary Table 1a: Primers for ChIP qPCR

Abbreviation	Forward primer (5'-3')	Reverse primer (3'-5')
**Acacb**	CAGGCAGCGAGCATTTCCTA	TCTGATGCCCTTGTGCCTAC
**Apoa4**	TCACTGGGGTGGAAAGAGGA	CCTGAACAGAACTGAGGCCC
**Cyp2a22**	AAGGCCATCATGTACCTGGC	TGGCATGGATCTACAAAGGCT
**Cyp2c39**	GGGTTACTCAACGATGCTCAA	TTGTGATCAGGCATCACTGGC
**Dhcr7**	CCTGCGTAGCTTGGTTTCCTA	CAGAAGCTGGGCTATGACGG
**Dhcr24**	CTGGATGCCCTGTGAGTTCTA	ACAGGCATTCGCAAACATACT
**G6pc**	CAGGACGAAGGGAGAGAGC	AGTTCAGGCTGAGGACCTTG
**Gck**	GCTGTGGGTGAGGACTGTCT	TTTAGGAGCCACCTCTCAGACT
**Gpam**	ACACACAAGGAGGAGTGCAG	CACGGTTGCCCAATGAGTTA
**Hmgcr**	GGCCGCCAATAAGGAAGGAT	GGAGACCGTTCGTGACGTAG
**Hmgcs1**	TGGTCGGAGAACCTCTCACT	CGAGAACAAGCCTGCCAATG
**Igfbp1**	CAGCACTTTCCACCGTTGAC	GCAGCTCCAGAGTTAGGCAA
**Pck1**	CAGGGCTGTCCTCCCTTCTA	CTGTTGACCGAGGGTGTGTT
**nCtrl**	GCTGGCAGAATAGCATCCG	TGATGAAGCACTCGTTGAGGC

Supplementary Table 1b: Primers for mRNA

Abbreviation	Forward primer (5'-3')	Reverse primer (3'-5')
**Acacb**	CCTTTGGCAACAAGCAAGGTA	AGTCGTACACATAGGTGGTCC
**Apoa4**	CGTGGACCTGCAAGATCAGA	TCTGCATGCGCTGGATGTAT
**Ccl2**	TTAAAAACCTGGATCGGAACCAA	GCATTAGCTTCAGATTTACGGGT
**Cyp2a22**	GTCACTCGCCTCTGCAAAAC	TGTACACTGGGCTTGGGAAC
**Cyp2c39**	GAGGAAGCATTCCAATGGTAGAA	TGTGAAGCGCCTAATCTCTTTC
**Dhcr7**	AGCTTCAGGCAGGCACTTAG	TGCTGGGATTTCGAAGCCAT
**Dhcr24**	CTGAAGACAAACCGGGAGGG	AAGATGGGGTTGTTGCCGAA
**E12**	TGCAGGATGAGCAGTTTGGT	GAGGCCTTTAAGGAGCTCGG
**E47**	TTATCCGACTTGAGGTGCAG	CTGGAGGAGAAGGACCTGAG
**Gck**	AACGACCCCTGCTTATCCTC	CTTCTGCATCCGGCTCATCA
**Gpam**	CTTCTAAGTCACCCACACCTGG	TTGCTTACTGGTCCTGTATCCT
**Gilz**	ACCACCTGATGTACGCTGTG	TCTGCTCCTTTAGGACCTCCA
**G6pc**	CGACTCGCTATCTCCAAGTG	GTTGAACCAGTCTCCGACC
**Hmgcr**	AGCTTGCCCGAATTGTATGTG	TCTGTTGTGAACCATGTGACTTC
**Hmgcs1**	GATCCCCTTTGGTGGCTGAAG	GAAAGAGCTGTGTGAAGGACAG
**Il6**	TAGTCCTTCCTACCCCAATTTCC	TTGGTCCTTAGCCACTCCTTC
**Igfbp1**	TCGTGACCACTGAGCAACTG	AGTTAGGAACTCGGGCATCG
**Pck1**	CTGCATAACGGTCTGGACTTC	CAGCAAACTCCCGTACTCC
**U36b4**	AGATTCGGGATATGCTGTTGGC	TCGGGTCCTAGACCAGTGTTC
