# Supplementary material for: E47 modulates hepatic glucocorticoid action
Source: Nat Commun. 2019 Jan 18;10:306. doi: 10.1038/s41467-018-08196-5 (PMC6338785; doi:10.1038/s41467-018-08196-5)
Supplement: Supplementary file 3 — Description of Additional Supplementary Files [file 41467_2018_8196_MOESM3_ESM.docx]

Description of Additional Supplementary Files

**File Name:** Supplementary Data 1

**Description:** ChIP-Seq data. GR and E2A ChIP-Seq peaks from Dex-treated mouse liver. Reproducible, annotated peaks called in 2 biological replicates are listed by chromosomal position.

**File Name:** Supplementary Data 2

**Description:** RNA-Seq data analyses from different tissues. Genes differentially expressed in liver, muscle or WAT of untreated and Dex/Corttreated E47 mutant mice. Values are fold change in gene expression as log2 (log2FC).

**File Name:** Supplementary Data 3

**Description:** Relative luciferase activity. Luciferase activity for each human reporter sequence, normalized to transfection efficiency, to vehicle and to empty vector. Averages of triplicates are shown.
